# Influence of Soil Moisture on Litter Respiration in the Semiarid Loess Plateau

**DOI:** 10.1371/journal.pone.0114558

**Published:** 2014-12-04

**Authors:** Yanjun Zhang, Shengli Guo, Qingfang Liu, Jishao Jiang

**Affiliations:** 1 State Key Laboratory of Soil Erosion and Dry-land Farming on the Loess Plateau, Institute of Soil and Water Conservation, Northwest A&F University, Yangling, China; 2 Institute of Soil and Water Conservation, Chinese Academy of Sciences and Ministry of Water Resource, Yangling, China; 3 College of Resources and Environment, Northwest A&F University, Yangling, China; University of Maryland, United States of America

## Abstract

Understanding the response mechanisms of litter respiration to soil moisture in water-limited semi-arid regions is of vital importance to better understanding the interplay between ecological processes and the local carbon cycle. *In situ* soil respiration was monitored during 2010–2012 under various conditions (normal litter, no litter, and double litter treatments) in a 30-year-old artificial black locust plantation (*Robinia pseudoacacia* L.) on the Loess Plateau. Litter respiration with normal and double litter treatments exhibited similar seasonal variation, with the maximum value obtained in summer (0.57 and 1.51 μmol m^−2 ^s^−1^ under normal and double litter conditions, respectively) and the minimum in spring (0.27 and 0.69 μmol m^−2 ^s^−1^ under normal and double litter conditions, respectively). On average, annual cumulative litter respiration was 115 and 300 g C m^−2^ y^−1^ under normal and double litter conditions, respectively. Using a soil temperature of 17°C as the critical point, the relationship between litter respiration and soil moisture was found to follow quadratic functions well, whereas the determination coefficient was much greater at high soil temperature than at low soil temperature (33–35% vs. 22–24%). Litter respiration was significantly higher in 2010 and 2012 than in 2011 under both normal litter (132–165 g C m^−2^ y^−1^ vs. 48 g C m^−2^ y^−1^) and double litter (389–418 g C m^−2^ y^−1^ vs. 93 g C m^−2^ y^−1^) conditions. Such significant interannual variations were largely ascribed to the differences in summer rainfall. Our study demonstrates that, apart from soil temperature, moisture also has significant influence on litter respiration in semi-arid regions.

## Introduction

In forests, above-ground litter plays an important role in controlling soil erosion, determining nutrient cycling, and improving the ecological environment [1], [2], [3], [4]. Litter can be decomposed via microbial respiration and it also leads to the accumulation of soil organic carbon (SOC) through humification [5], [6]. Environmental conditions are highly influential in determining whether litter will be a source for a soil carbon pool, as a greater proportion of litter may be humified under sub-optimal conditions such as cold temperatures or excessive moisture; otherwise, it may be decomposed and released into the atmosphere as CO_2_ [7]. Litter respiration is a major component of soil respiration in the carbon cycle of terrestrial ecosystems [8], [9]. Extensive investigations have been conducted around the world [10], [11], [12], with studies focusing mainly on tropical regions [13], [14], subtropical regions [12], [15], and cold temperature regions [16], [17], as well as natural forest ecosystems [11], [8], [14], [18]. However, information on temperate plantations is relatively scarce.

Extensive research has corroborated the fact that soil temperature is the most moderate abiotic factor driving the rate of respiration [8], [11], [19], although soil temperature alone cannot adequately explain spatial and temporal variations in respiration rate [11], [20], [21]. In addition to soil temperature, soil moisture also contributes to this variation, especially in water-limited ecosystems, because soil moisture is a critical environmental determinant limiting the response of ecological processes to temperature [11], [22], [23], [24]. For instance, in a Mediterranean mixed-oak forest ecosystem, the respiration rate was found to be highly correlated with soil temperature when volumetric soil moisture content was above 20%, but not when this was below 20% [11]. In Spain, respiration rate was shown to be largely dependent on soil temperature above a soil water content threshold value of 10% in forest and olive groves, and 15% in abandoned fields; below these thresholds, the respiration rate was mainly affected by soil moisture [22]. However, the response of respiration rate to soil moisture is extremely complex and may be confused with the effect of soil temperature. For example, in a semi-arid steppe ecosystem in Spain, soil moisture was the controlling driver of respiration rate for most of the year when temperatures were above 20°C, and it constrained the response to temperature for the few months when temperature was below 20°C [23]. Furthermore, the respiration rate can be lowered under both low and high soil moisture conditions by limiting the diffusion of available substrates and the diffusion of oxygen in soil [23], [25]. Notwithstanding the above, however, the rate of respiration, especially litter respiration in water-limited regions, has received comparatively little research attention [22].

The Loess Plateau of northwest China covers an area of 640,000 km^2^. It is an arid and semi-arid region, with a continental monsoon climate. In addition, the loess region is particularly susceptible to soil erosion because of its fractured and steep terrain, aggravated by improper land use. To address this problem, the black locust (*Robinia pseudoacacia* L.) has been widely planted in the region since 1980 to control soil erosion (due to its high drought resistance), now covering an area within the region of 70,000 ha. Moreover, in degraded dryland forest ecosystems such as this, above-ground litter decomposition has important practical implications for ecological rehabilitation [4], [6], even if the local population is used to collecting above-ground litter for heating and cooking. However, soil respiration inevitably varies with different litter treatments [8], [13], [26], [27], [28] through changes in the available substrate [29], [30], shifts in the size and community composition of micro-organisms [27], or through influencing soil microclimate, especially soil moisture [30], [31], [32]. To our knowledge, while a large number of studies have focused on the effect of above-ground litter on reducing soil erosion and improving the physical and chemical properties of the soil in this region [2], [4], there is scarce information concerning litter respiration and factors that influence it in this region.

Given the above, in this study we monitored *in situ* soil respiration and soil microclimate (temperature and moisture) in a 30-year-old artificial black locust plantation (*Robinia pseudoacacia* L.) within a small watershed; monitoring was conducted with different above-ground litter treatments over the course of three years (2010−2012). Litter treatments included normal litter, double litter, and no litter. The objectives of the study were: 1) to describe the dynamics of litter respiration with normal and double litter, and 2) to investigate the effect of soil moisture on litter respiration with normal and double litter in the semi-arid Loess Plateau.

## Materials and Methods

### 2.1 Ethics Statement

There were no specific permits required for the described field studies. We confirmed that the site was not privately owned or protected in any way. The field studies did not involve endangered or protected species.

### 2.2 Site description

The study site was located on the southern Loess Plateau, at Wang-donggou watershed, Changwu, Shaanxi, China, and forms part of the Changwu State Key Agro-Ecological Experimental Station, Chinese Academy of Sciences (35°13′ N, 107°40′ E; 1095 m above sea level). The study site has a continental monsoon climate, with mean annual rainfall of 580 mm and mean annual air temperature of 9.4°C. Rainfall variation occurs both seasonally and annually, and more than 50% of rainfall falls in summer (between June and August). Air temperature variations occur only seasonally, as the monthly mean temperature in summer is much higher than in other seasons. Site characteristics include the following: ≥10°C accumulated temperature of 3029°C, annual sunshine duration of 2230 h, annual total radiation of 484 kJ cm^–2^, and a frost-free period of 171 days.

Soils of interest were derived from wind-deposited loess and form part of the loessial soil group, according to the soil classification system of the Food and Agriculture Organization and the United Nations Educational Scientific and Cultural Organization (FAO-UNESCO). The parent material was relatively uniform calcareous loess dominated by loam. Soil collected in 2009 had a depth of 0–20 cm, a pH of 8.3, a clay content (<0.002 mm) of 24%, a field capacity of 22.4%, a permanent wilting point of 9.0%, an SOC level of 6.80 g·kg^−1^, total nitrogen (TN) of 0.66 g·kg^−1^, and an initial NaHCO_3_-extractable soil phosphorus content of 5 mg P kg^−1^.

### 2.3 Plantation description

The black locust plantation has a tree density of 1213 stems ha^−1^ and was established approximately 30 years ago to control soil erosion and improve the ecological environment of the region. Approximately 64% of the study site is covered with trees, with these having a total area of 0.68 ha. Shrubs cover 24%, and the remaining 12% is covered by grass and bare soil. The plantations are located on a ridge with a gentle slope (<15°) on the west of the Loess plateau. Tree canopy height (H) and diameter at breast height (DBH, 1.3 m above the ground) were recorded for trees with DBH>1 cm. Mean H was 6.8±1.6 m, mean DBH was 6.4±2.6 cm, and the canopy area was 55%. The main understory shrub was *Rubus parvifolius* L., with a mean H of 62.8±11.8 cm and a canopy area of 55%. The main understory herb was *Bothriochloa ischaemum* (L.) Keng, with a mean H of 42.5±11.9 cm and a canopy area of 75%.

### 2.4 Litter treatments and management

Slope tillage was converted to woodland with the implementation of integrated management of small watersheds in the 1980s to control soil erosion, because local people collected forest above-ground litter for heating and cooking. In March 2009, we chose a relatively homogeneous black locust plantation as a study site, and established an experiment with three litter treatments (1.5 m×1.5 m; three replicates per treatment) at the site. For the normal litter treatment, litter inputs were allowed. For the no litter treatment, litter inputs were prevented using a sheet of nylon mesh screen (pore size ∼1.7 mm) over the exposed soil area. The protective screen was temporarily removed to allow for soil respiration measurements but otherwise left intact over the study period. For the double litter treatment, above-ground litter inputs were doubled by adding litter from no litter plots four to five times per year. Large branches that fell on screens were discarded. Five 1 m×1 m nylon meshes, with a mesh size of 1 mm×1 mm, were placed in the black locust plantation from 2010 to 2012 to collect above-ground litter. Litter was collected seasonally and dried at 70°C for 72 h in an oven. Mean annual above-ground litter content was 474 g m^−2^.

Litter respiration under normal litter conditions was determined as the difference in soil respiration between normal and no litter treatments, while litter respiration with double litter was defined as the difference in soil respiration between double litter and no litter treatments.

The litter contribution rate (%) was determined by dividing litter respiration under normal litter by soil respiration under normal litter.

### 2.5 Measurements of soil respiration, soil temperature, and moisture

Soil respiration was measured using an automated closed soil CO_2_ flux system equipped with a portable chamber (20 cm in diameter; Li-8100, Lincoln, NE, USA). Approximately one day before the first measurement, a polyvinyl chloride (PVC) collar (20 cm in diameter by 12 cm in height) was inserted 2 cm into each plot, where it remained for the entire experimental period (from 2010 to 2012). Before measurements were taken, all visible living flora and fauna were removed. If necessary, one or more additional measurements were taken until the variation between two consecutive measurements was less than 15% for a given collar. Final soil respiration values for a given collar were calculated as the mean values of two consecutive satisfactory measurements, with a 30 s delay between measurements. The measurement time for each collar was 150 s, which included 30 s pre-purge, 30 s post-purge, and a 90 s observation period. Field measurements were conducted between 09∶00 and 11∶00 a.m. from March 2010 to November 2012 (but not in December, January, or February because of cold weather). A total of 12, 15, and 19 soil respiration measurements were taken in 2010, 2011, and 2012, respectively.

Soil temperature (three measurements per collar) and moisture (four measurements per collar) were measured 10 cm away from the collar chamber; soil respiration was measured at the same time. Soil temperature and moisture at 5 cm depth were measured using a Li-Cor thermocouple probe and a Theta Probe ML2X with an HH2 moisture meter (Delta-T Devices, Cambridge, UK), respectively. The soil water-filled pore space (WFPS) was calculated using the following equation: WFPS (%) = (volumetric water content/100×[2.65–soil bulk density]/2.65).

### 2.6 Data analysis

Respiration rate, soil temperature, and soil moisture data were analyzed using the GLM procedure in SAS to detect any differences (ANOVA test at *P* = 0.05) between different litter treatments. An exponential function was used to describe the relationship between soil respiration and soil temperature [33]. Our method for determining litter respiration sometimes led to negative litter respiration under normal and double litter conditions, which might have resulted from high variability in respiration rates relative to a low treatment effect [20], or they might indirectly reflect the response of soil respiration to soil moisture. Negative litter respiration values under normal and double litter treatments were retained in the data sets and incorporated into statistical models; thus, a linear increased function was used to describe the relationship between litter respiration and soil temperature [20]. Additionally, an exponential function was used to describe the relationship between litter respiration (after removing negative litter respiration values under normal and double litter treatments) and soil temperature [33]. A quadratic regression function was used to describe the relationship between respiration and soil moisture [25]. Additionally, respiration can be more accurately simulated when considering the interplay between soil temperature and moisture content than when considering either soil temperature or moisture in isolation [20], [34]. After comparing different functional forms and checking residual plots, a bivariate model was adopted to simulate the effect of soil moisture content and temperature on respiration as follows:
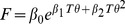
(1)where T is soil temperature at 5 cm depth, *θ* is soil moisture at 0–5 cm depth, and *β*_0_, *β*_1_, and *β*_2_ fitted parameters.

Annual cumulative litter respiration was estimated by interpolating between measurement dates to obtain the mean daily respiration for each plot (negative litter respiration values were replaced with a value of zero), and then summing mean daily respiration for a given year.

## Results

### 3.1 Effect of litter treatments on soil temperature and moisture

**Figure 1:**
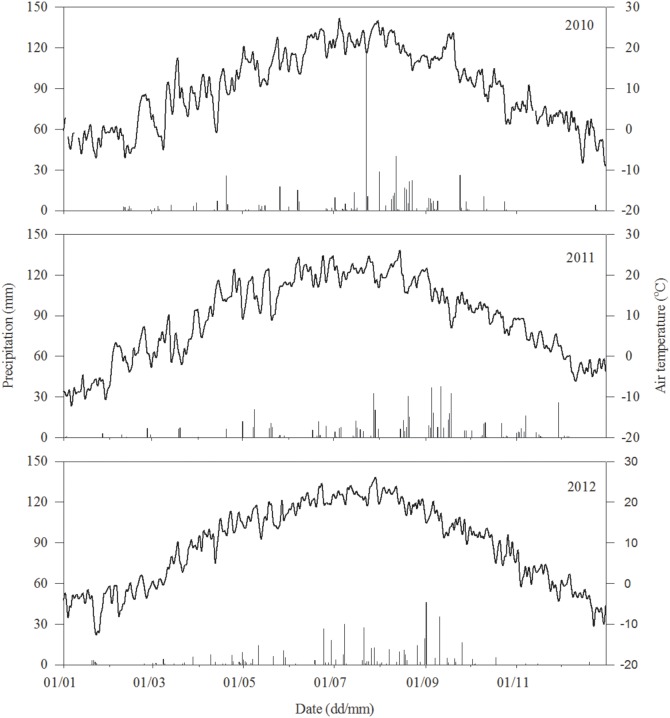
Variation in rainfall (mm) and air temperature (°C) in 2010, 2011, and 2012, respectively.

**Figure 2:**
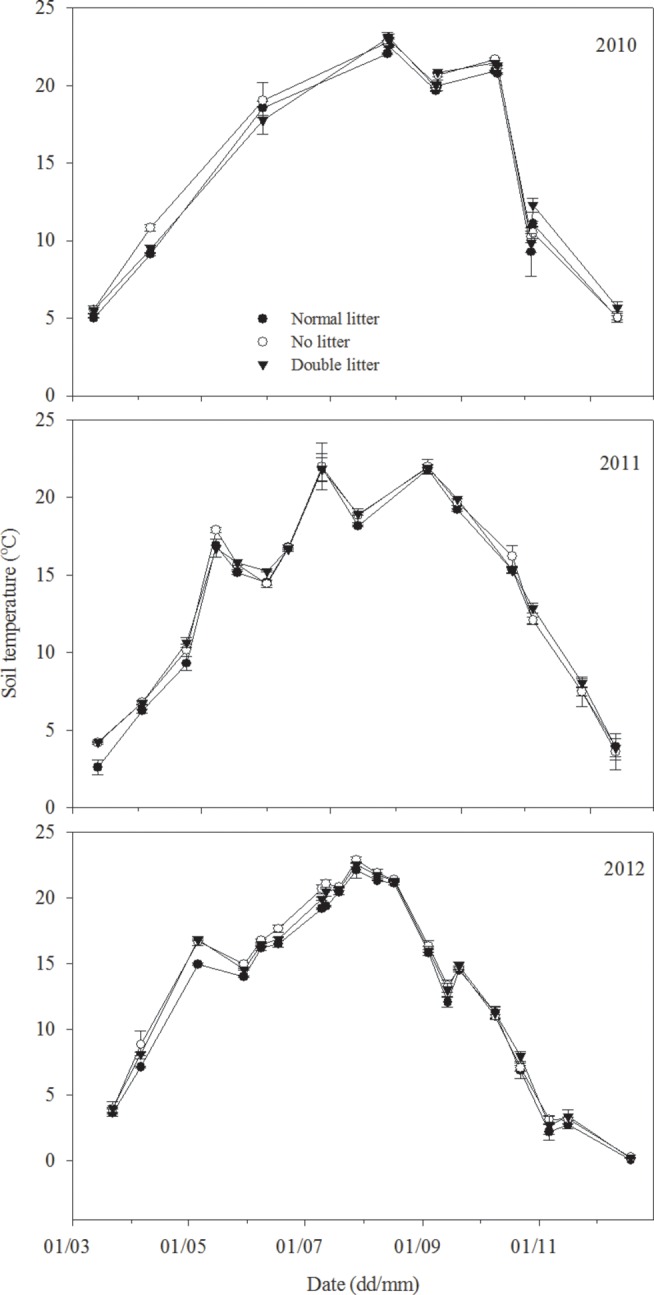
Variation in soil temperature (°C) at a depth of 5 cm for different litter treatments, including normal litter, no litter, and double litter treatments in 2010, 2011, and 2012, respectively. The asterisk indicates a significant difference at *P*<0.05.

The soil temperature at 5 cm depth exhibited seasonal variation, with a similar trend under normal litter, no litter, and double litter treatments (*P*>0.05). The patterns of soil temperature variation followed variations in air temperature, with the lowest soil temperatures recorded in the spring and autumn and the highest in summer (Figs. 1 and 2). Mean soil temperature was as follows: no litter treatment (14.75°C) > double litter treatment (14.70°C) > normal litter treatment (14.17°C).

**Figure 3:**
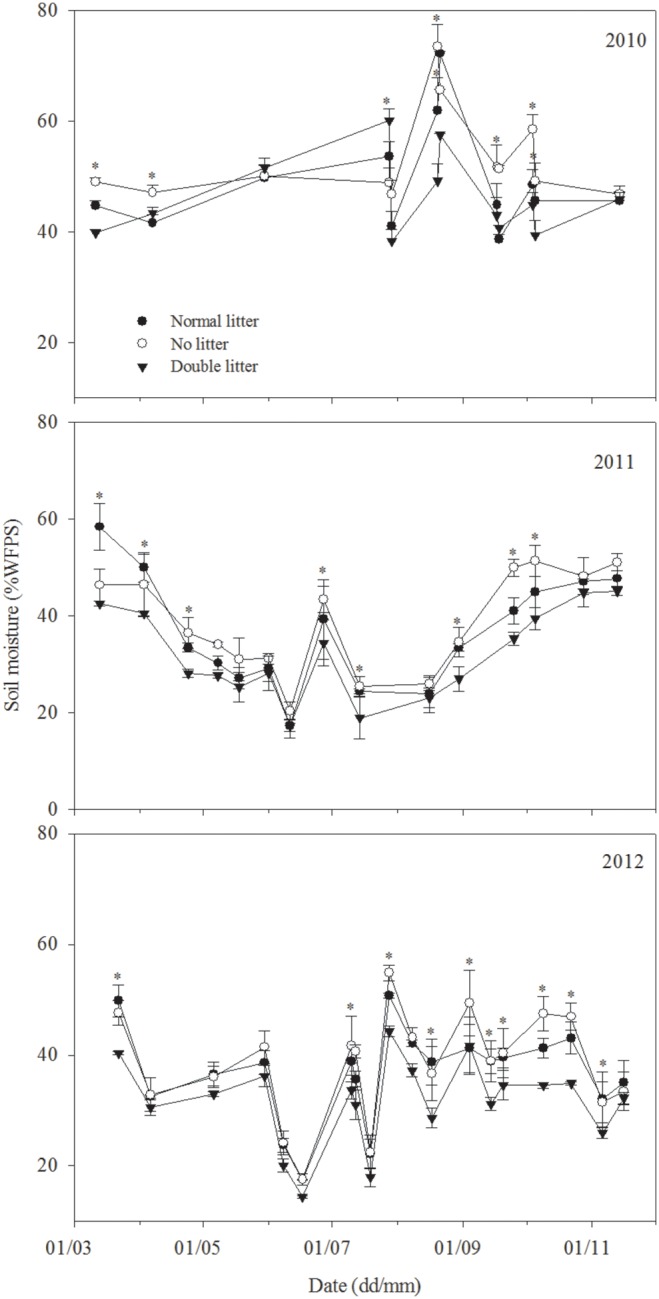
Variation in soil moisture (% WFPS) at a depth of 0−5 cm for different litter treatments, including normal litter, no litter, and double litter treatments in 2010, 2011, and 2012, respectively. The asterisk indicates a significant difference at *P*<0.05.

Soil moisture in the 0–5 cm layer also exhibited seasonal variation, with a similar order of variations between treatments as above; the difference between treatments was also found to be statistically significant (*P*<0.05) (Fig. 3). Mean soil moisture was shown to be as follows: no litter treatment (43.28% WFPS) > normal litter treatment (40.74% WFPS) > double litter treatment (36.55% WFPS) (Fig. 3).

### 3.2 Effect of litter treatments on soil respiration

**Figure 4:**
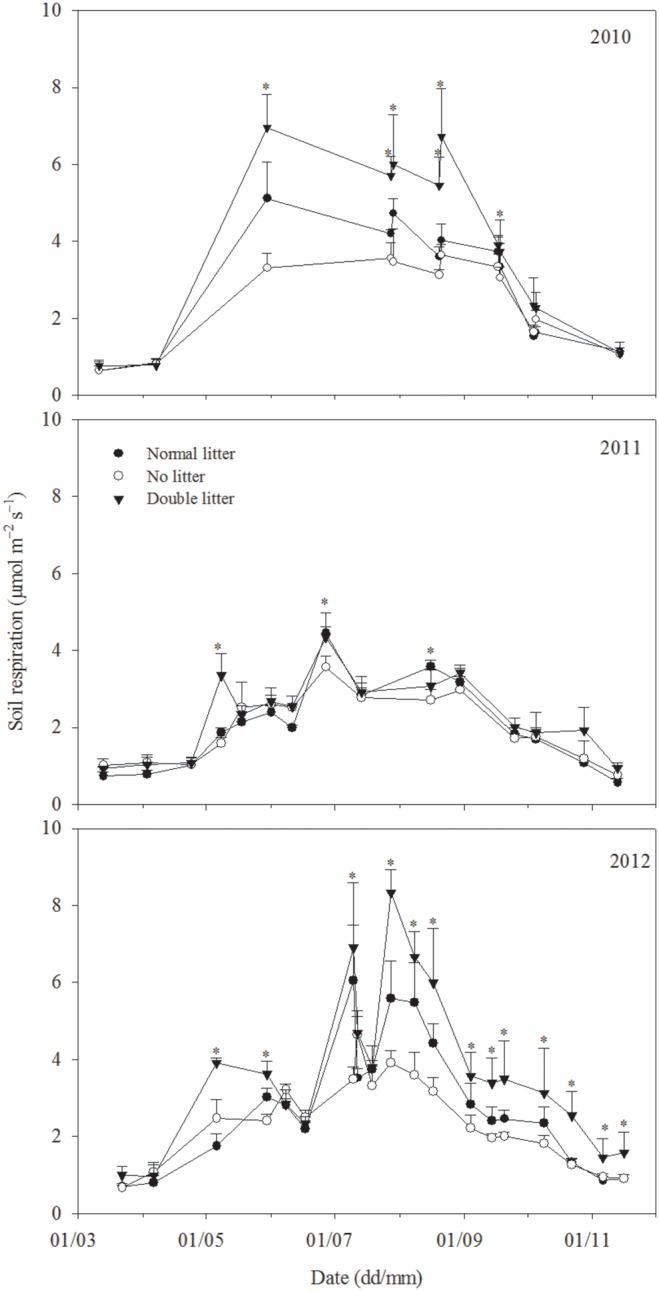
Variation in soil respiration (μmol m^−2 ^s^−1^) under normal litter, no litter, and double litter treatments in 2010, 2011, and 2012, respectively. The asterisk indicates a significant difference at *P*<0.05.

Soil respiration across the three treatments exhibited similar seasonal variation, with patterns of variation corresponding to variations in temperature; the maximum value occurred in summer and the minimum value was recorded in the spring or autumn (Figs. 1 and 4). Mean soil respiration under normal, no litter and double litter treatments was 0.50–6.64 μmol m^−2 ^s^−1^, 0.62–4.43 μmol m^−2 ^s^−1^, and 0.73–8.84 μmol m^−2 ^s^−1^ over three years (2010–2012), respectively.

With a double litter treatment, soil respiration increased by 33%, 12%, and 30% in 2010, 2011, and 2012, respectively. Under a no litter treatment, soil respiration decreased by 14%, 2%, and 15% over the same three years, respectively. Mean annual cumulative soil respiration was 777 g C m^−2^ y^−1^, 657 g C m^−2^ y^−1^, and 967 g C m^−2^ y^−1^ under normal litter, no litter, and double litter treatments, respectively (Table 1). Additionally, annual mean cumulative soil respiration decreased by 18% with no litter, and increased by up to 24% with double litter, compared with normal litter conditions (Table 1).

**Table 1:** Annual soil respiration (g C m^−2^ y^−1^), annual litter respiration (g C m^−2^ y^−1^), annual litter contribution rate (%), and summer rainfall (mm) from 2010 to 2012.

Year	Soil respiration			Litter respiration		Littercontributionrate	Summerrainfall
	Normallitter	Nolitter	Double litter	Normal litter	Double litter		
2010	950±73a	766±42b	1151±111c	165±34a	418±25b	19±7	385
2011	635±40a	529±37b	724±36c	48±19a	93±17b	8±6	220
2012	745±73a	675±37a	1025±192b	132±21a	389±30b	17±4	241

Values are expressed as mean ± SE. Different letters following the data in the same row denote a significant difference at *P*<0.05 levels.

### 3.3 Variation in litter respiration

**Figure 5:**
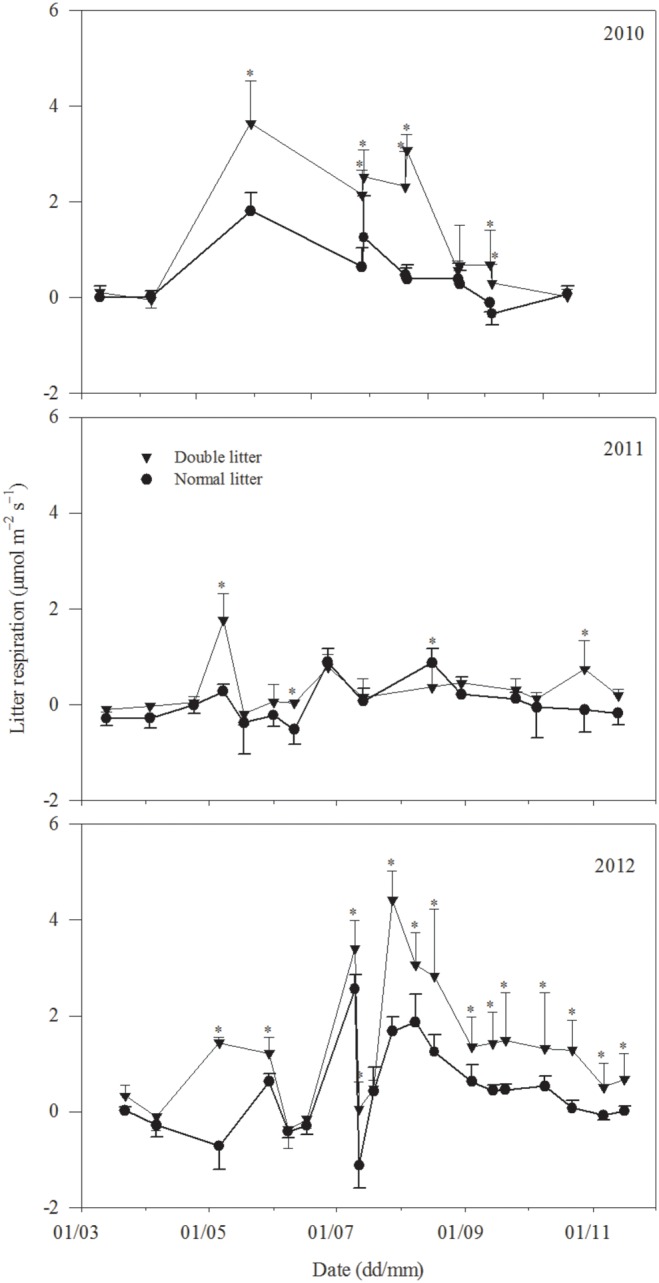
Variation in litter respiration (μmol m^−2 ^s^−1^) with normal and double litter treatments in 2010, 2011, and 2012, respectively. The asterisk indicates a significant difference at *P*<0.05.

Litter respiration exhibited similar seasonal variations with normal and double litter treatments and variation patterns corresponded with variations in temperature, with the maximum value occurring in summer and the minimum value occurring in spring or autumn (Figs. 1 and 5). Annual mean litter respiration under the normal treatment was 0.41 μmol m^−2 ^s^−1^, 0.06 μmol m^−2 ^s^−1^, and 0.61 μmol m^−2 ^s^−1^ in 2010, 2011, and 2012, respectively. Under double litter conditions, values for the same parameter were 1.33 μmol m^−2 ^s^−1^, 0.30 μmol m^−2 ^s^−1^, and 1.49 μmol m^−2 ^s^−1^ over the three years. Litter respiration with normal and double litter treatments was closely related to soil microclimate (Tables 2, 3, and 4); it was higher in summer due to heavy rainfall and higher temperatures, and lower in other seasons due to less rainfall and lower temperatures (Figs. 1 and 5). For example, litter respiration under normal litter conditions was 0.69 μmol m^−2 ^s^−1^ in summer but only 0.27 μmol m^−2 ^s^−1^ in other seasons; similarly, litter respiration with double litter in 2010 was 2.51 μmol m^−2 ^s^−1^ in summer but only 0.74 μmol m^−2 ^s^−1^ in other seasons. A similar trend was observed in 2011 and 2012.

**Table 2:** An exponential function was used to describe the relationship between soil respiration (F) and soil temperature (T) under normal litter, no litter, and double litter treatments.

Year	Soil respiration									Litter respiration						
	Normallitter			No litter			Doublelitter			Normallitter			Doublelitter			
	Functions	*R^2^*	*P*	Functions	*R^2^*	*P*	Functions	*R^2^*	*P*	Functions	*R^2^*	*P*	Functions	*R^2^*	*P*	
2010	F = 0.67e^0.087 T^	0.84	<0.01	F = 0.70e^0.073 T^	0.89	<0.01	F = 0.94e^0.081 T^	0.68	<0.01	F = 0.05 T–0.44	0.57	<0.05	F = 0.13 T–0.72	0.45	<0.05	
2011	F = 0.44e^0.100 T^	0.93	<0.01	F = 0.78^e0.065 T^	0.70	<0.01	F = 0.74e^0.075 T^	0.84	<0.01	F = 0.04 T–0.56	0.40	<0.05	F = 0.02 T–0.18	0.35	<0.05	
2012	F = 0.59e^0.100 T^	0.84	<0.01	F = 0.69e^0.078 T^	0.90	<0.01	F = 0.80e^0.095 T^	0.79	<0.01	F = 0.08 T–0.55	0.42	<0.05	F = 0.12 T–0.29	0.34	<0.05	

A linear function was used to describe the relationship between litter respiration (F) and soil temperature (T) under normal and double litter treatments. Values are from 2010 to 2012 at 95% confidence intervals; the values were calculated using a procedure of REG of SAS.

**Table 3:** An exponential function was used to describe the relationships between litter respiration (F) and soil temperature (T) with normal and double litter treatments after removing negative litter respiration values.

Year	Normal litter			Double litter		
	Functions	*R^2^*	*P*	Functions	*R^2^*	*P*
2010	F = 0.0012e^0.1898 T^	0.59	<0.05	F = 0.0334e^0.1875 T^	0.67	<0.05
2011	F = 0.0002e^0.1541 T^	0.68	<0.01	F = 0.0569e^0.1076 T^	0.34	<0.05
2012	F = 7.90e^0.1423 T^	0.83	<0.01	F = 0.4247e^0.0804 T^	0.54	<0.05

Values are from 2010 to 2012 at 95% confidence intervals; values were calculated using a procedure of REG of SAS.

**Table 4:** A quadratic regression function was used to describe the relationship between soil respiration (F), soil moisture (*θ*), and litter respiration (F), under normal, no litter, and double litter treatments.

Treatment		LT^*^			HT^*^		
		Functions	*R^2^*	*P*	Functions	*R^2^*	*P*
Soil respiration	Normal litter	F = –0.0009*θ* ^2^+0.03*θ*+1.85	0.24	<0.05	F = –0.0016*θ* ^2^+0.16*θ*+0.27	0.33	<0.05
	No litter	F = 0.0019*θ* ^2^–0.19*θ*+5.90	0.28	<0.05	F = –0.000*θθ* ^2^+0.06*θ*+1.69	0.40	<0.05
	Double litter			>0.05	F = –0.0024*θ* ^2^+0.26*θ*–0.79	0.41	<0.05
Litter respiration	Normal litter	F = –0.0012*θ* ^2^+0.11*θ*–2.31	0.24	<0.05	F = –0.0015*θ* ^2^+0.14*θ*–2.25	0.33	<0.05
	Double litter	F = –0.0035*θ* ^2^+0.23*θ*–3.13	0.22	<0.05	F = –0.0017*θ* ^2^+0.19*θ*–2.85	0.35	<0.05

LT means data with soil temperature lower than 17°C, and HT means data with soil temperature higher than 17°C. Values are from 2010 to 2012 at 95% confidence intervals; values were calculated using a procedure of REG of SAS.

Litter respiration with normal and double litter treatments was significantly higher in 2010 and 2012 than in 2011. Annual cumulative litter respiration with normal and double litter treatments was 165 g C m^−2^ y^−1^ and 418 g C m^−2^ y^−1^ in 2010, 48 g C m^−2^ y^−1^ and 93 g C m^−2^ y^−1^ in 2011, and 132 g C m^−2^ y^−1^ and 389 g C m^−2^ y^−1^ in 2012, respectively (Table 1). Thus, mean annual cumulative litter respiration under normal litter conditions was 115 g C m^−2^ y^−1^; with double litter treatment this increased to 300 g C m^−2^ y^−1^, respectively. Annual litter contribution rate was 19%, 8%, and 17% in 2010, 2011, and 2012, respectively, with a mean value of 15% (Table 1).

### 3.4 Response of litter respiration to soil microclimate

Soil respiration under the three treatments increased exponentially with soil temperature (Table 2), with almost significant influence of soil moisture, as shown by a quadratic equation; the exception was soil respiration with the double litter treatment at relatively lower temperatures (i.e., when soil temperature was less than 17°C) (Table 4).

Litter respiration with normal and double litter treatments increased linearly with soil temperature throughout (Table 2); however, it also increased exponentially with soil temperature after removal of negative litter respiration values for normal and double litter treatments (Table 3). Litter respiration under the latter two treatments was also significantly influenced by soil moisture, as shown by a quadratic worked out after measurement data were partitioned into two subsets using a soil temperature of 17°C as the critical point (Table 4).

## Discussion

### 4.1 Soil respiration in water-limited region

Annual cumulative soil respiration across three treatments ranged from 529–1151 g C m^−2^ y^−1^ over the different years, with these values falling with the range of global mean cumulative soil respiration values (160–2450 g C m^−2^ y^−1^) for forest ecosystems [35], [36]. However, mean annual soil respiration rate in our study (2.47–3.00 µmol m^−2 ^s^−1 ^g) was much lower than in the deciduous forests of the northern hemisphere temperate regions (3.5 µmol m^−2 ^s^−1^) [37]. This is because the study site forms part of a degraded ecosystem, characterized by lower ecosystem productivity due to long-standing soil erosion. Comparatively less rainfall may be another reason for the lower values recorded. Annual cumulative soil respiration under normal litter, no litter, and double litter treatments ranged from 635 to 950 g C m^−2^ y^−1^, 529 to 766 g C m^−2^ y^−1^, and 724 to 1151 g C m^−2^ y^−1^, respectively. Inter-annual variation may be explained by differences in summer precipitation (Table 1).

Soil respiration across three treatments exhibited similar seasonal variation (Fig. 4), mainly influenced by soil temperature, with exponential increasing functions (Table 2); similar results were also obtained in other studies [19], [33], [38]. Interestingly, soil temperature alone cannot adequately explain this variation (Table 2). Soil moisture may be another influential abiotic factor, especially in semi-arid regions [22], [24]. However, the response of soil respiration to soil moisture is controversial, involving complex mechanisms [25], [39], [40]. These complex mechanisms may be confused with the effect of soil temperature [23], [37]. To address this issue, measurement data were partitioned into two subsets using a soil temperature of 17°C as the critical point. Results showed that soil respiration was almost significantly influenced by soil moisture with a quadratic (albeit to a lesser degree), with the exception of soil respiration under double litter treatment at relatively lower temperatures (when soil temperature was less than 17°C) (Table 4). Similar results have also been recorded for semi-arid steppe ecosystems of Spain, where soil moisture is the controlling driver of soil respiration for most of the year when temperatures are above 20°C, also constraining the response to temperature for the few months when temperature is below 20°C [23].

### 4.2 Influence of soil moisture on litter respiration

The range of annual cumulative litter respiration with normal and double litter treatments recorded in our study site between 2010 and 2012 lies within the documented range of annual cumulative soil heterotrophic respiration data (61–970 g C m^−2^ y^−1^) [41], [42]. However, these values (115 and 300 g C m^−2^ y^−1^ for annual cumulative litter respiration with normal and double litter treatments) are much lower than values measured in forest systems elsewhere having adequate rainfall [8], [15], [28]. We speculate that this difference may be attributed to low rainfall (580 mm vs. 1925 mm). Additionally, the lower litter biomass in our site (474 g m^−2^ y^−1^ vs. 627 g m^−2^ y^−1^) also contributed to this difference [10], [12], [15]. Annual cumulative litter respiration under normal and double litter treatments ranged from 48–165 g C m^−2^ y^−1^, and 93–418 g C m^−2^ y^−1^, respectively; this inter-annual variation may be ascribed to differences in summer precipitation (Table 1).

**Figure 6:**
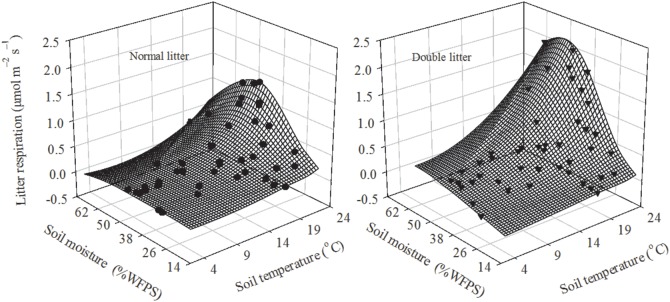
Response surface of litter respiration with normal and double litter treatments as a function of soil moisture and soil temperature from 2010 to 2012.

Litter respiration with normal and double litter treatments exhibited similar seasonal variation (Fig. 5), which was significantly greater in summer (June to August) due to heavy rainfall and higher temperatures than in other seasons with less rainfall and lower temperatures (0.64 μmol m^−2 ^s^−1^ vs. 0.06 μmol m^−2 ^s^−1^ under normal litter conditions, and 1.59 μmol m^−2 ^s^−1^ vs. 0.63 μmol m^−2 ^s^−1^ under double litter conditions). Similar results were also noted in DIRT (Detritus Input and Removal Treatments) experiments conducted at the H.J. Andrews Experimental Forest, Oregon [8]. Seasonally, litter respiration with normal and double litter was mainly impacted by soil temperature, with linear increased functions (Table 2). Similar results were obtained in studies at the Priest River Experimental Forest in the northern Rocky Mountains, USA [20]. Moreover, litter exacerbated the effect of drought stress on soil respiration and we obtained negative litter respiration values under normal and double litter treatments in seasons with lower rainfall over the course of the three years of the study (Fig. 5). After removing these negative values from all data sets, we found that litter respiration with normal and double litter treatments increased exponentially with soil temperature (Table 3). However, soil temperature can only explain 40–57% and 34–54% of variation under normal and double litter treatments, respectively, with linear increased functions for all the data (Table 2). Similarly, 49%–66% of the variation in litter respiration under normal conditions and 34%–67% of the variation under double litter conditions could be explained in this manner, with exponentially increasing functions after removal of these negative litter respiration values (Table 3). Similar results are also found in other studies [11], [20], [21]. In addition to soil temperature, soil moisture also contributes to larger seasonal variations in litter respiration under normal and double litter treatments through its significant fluctuations (both seasonally and annually) due to extremely uneven rainfall distribution, which is particularly significant in water-scarce arid and semi-arid regions [22], [24]. However, the response of litter respiration with normal and double litter treatments to soil moisture is complex, and we found that, in these cases, litter respiration was not correlated with soil moisture for all the data in a given year. More interestingly, litter respiration with normal and double litter treatments was significantly correlated with soil moisture with a quadratic using a soil temperature of 17°C as the critical point for partitioning measurement data (Table 4). Further analysis showed clearly that the determination coefficient (*R* ^2^) was lower at lower soil temperatures than at higher soil temperatures (24% vs. 33% for litter respiration under normal litter treatment, and 22% vs. 35% for litter respiration under double litter treatment) (Table 4). Similar results have also been obtained in the semi-arid steppe ecosystems of Spain, where soil moisture is the main driver of soil respiration for most of the year when temperatures are above 20°C [23]. More importantly, the response surface of litter respiration with normal and double litter treatments to integrated soil temperature and moisture (including both seasonal and annual variation) could definitely and intuitively describe the response of litter respiration under these treatments to the soil microclimate (Fig. 6). Similar results have also been obtained in the Priest River Experimental Forest in the northern Rocky Mountains, USA [20].

### 4.3 Effect of litter management practices on soil respiration

Soil respiration increased by 24% due to addition of litter, but decreased by 18% as a result of removing litter. Similar results were are also found in the previous studies [8], [26], [28]. The results presented herein may clearly exhibit a positive priming effect through the input of fresh litter-fall, which can be indirectly confirmed by the fact that when litter increased by a factor of two (normal litter vs. double litter) soil respiration increased 2.6 times (annual cumulative litter respiration under normal litter conditions was 115 g C m^−2^ y^−1^ but 300 g C m^−2^ y^−1^ with the double litter treatment); thus the priming effect cannot be ignored [11], [14], [30], [45]. Variations in soil respiration with different litter management practices can be explained as follows. First, different litter management practices imply different available substrates for soil micro-organisms [29], [30], with increased litter enhancing the available substrate and removal of litter reducing it [29]. Second, litter management practices caused shifts in the size and community composition of micro-organisms, such as the ratio of soil fungi to bacteria; increased litter increased the ratio and removing litter reduced it [43], [44], with the increase or decrease of the ratio related to having less or more available substrate [27]. Finally, litter management practices influence soil microclimate (temperature and moisture), thus indirectly mediating the activity and constitution of micro-organisms [30], [31], [32].

Mean annual cumulative litter respiration (litter respiration under normal litter) in our sites was 115 g C m^−2^ y^−1^, which was 15% of the total soil respiration. On the other hand, the carbon input into the soil through littering was 213 g C m^−2^ y^−1^ (based on the mean annual litter input of 474 g m^−2^ y^−1^ and a carbon content of 45%), implying that approximately 50% of organic carbon remained in the system. Apparently, the accumulated litter can not only effectively alleviate soil erosion, but can also progressively improve the ecological environment in the degraded and eroded loess regions [2], [4], [46], [47]. Therefore, litter conservation in the black locust plantation may be a win-win policy from the standpoint of increasing SOC storage and improving the ecological environment.

## Conclusions

Understanding the effect of soil moisture on litter respiration is of prime importance in water-limited regions in order to better understand the carbon cycle. Our study demonstrated that litter management had significant impacts on soil respiration, showing 24% increase but 18% decrease under litter addition and removal, respectively. Litter respiration with normal and double litter treatments exhibited similar seasonal variation, being controlled mainly by soil temperature; however, soil moisture also contributed to larger variations. Significant inter-annual variation in litter respiration was ascribed to differences in summer rainfall. Our study demonstrates that both soil temperature and moisture have significant influences on litter respiration in semi-arid regions.
